# Supplementary material for: A fault-intrusion-tolerant system and deadline-aware algorithm for scheduling scientific workflow in the cloud
Source: PeerJ Comput Sci. 2021 Nov 2;7:e747. doi: 10.7717/peerj-cs.747 (PMC8576554; doi:10.7717/peerj-cs.747)
Supplement: Supplemental Information 6 [file peerj-cs-07-747-s006.zip › Myproject1.1/docs/org/cloudbus/cloudsim/network/class-use/GraphReaderIF.html]

Uses of Interface org.cloudbus.cloudsim.network.GraphReaderIF


JavaScript is disabled on your browser.


- Overview
- Package
- Class
- Use
- Tree
- Deprecated
- Index
- Help

- Prev
- Next

- Frames
- No Frames

- All Classes

## Uses of Interface org.cloudbus.cloudsim.network.GraphReaderIF

- Packages that use GraphReaderIF

  | Package | Description |
  | --- | --- |
  |  |  |
  | --- | --- |
  | org.cloudbus.cloudsim.network |  |
- - ### Uses of GraphReaderIF in org.cloudbus.cloudsim.network

    Classes in org.cloudbus.cloudsim.network that implement GraphReaderIF

    | Modifier and Type | Class and Description |
    | --- | --- |
    |  |  |
    | --- | --- |
    | `class` | `GraphReaderBrite` This class is just an file-reader for the special brite-format! the brite-file is structured as followed: Node-section: NodeID, xpos, ypos, indegree, outdegree, ASid, type(router/AS) Edge-section: EdgeID, fromNode, toNode, euclideanLength, linkDelay, linkBandwith, AS\_from, AS\_to, type |

- Overview
- Package
- Class
- Use
- Tree
- Deprecated
- Index
- Help

- Prev
- Next

- Frames
- No Frames

- All Classes
